# Recent Strategies for Bioremediation of Emerging Pollutants: A Review for a Green and Sustainable Environment

**DOI:** 10.3390/toxics10080484

**Published:** 2022-08-19

**Authors:** Saroj Bala, Diksha Garg, Banjagere Veerabhadrappa Thirumalesh, Minaxi Sharma, Kandi Sridhar, Baskaran Stephen Inbaraj, Manikant Tripathi

**Affiliations:** 1 Department of Microbiology, Punjab Agriculture University, Ludhiana 141001, India; 2 Microbial Processes and Technology Division, CSIR-National Institute for Interdisciplinary Science and Technology, Thiruvananthapuram 695019, India; 3 Laboratoire de Chimie Verte et Produits Biobasés, Département Agro Bioscience et Chimie, Haute Ecole Provinciale de Hainaut-Condorcet, 11 Rue de la Sucrerie, 7800 Ath, Belgium; 4 UMR1253, Science et Technologie du Lait et de l’œuf, INRAE, L’Institut Agro Rennes-Angers, 65 Rue de Saint Brieuc, F-35042 Rennes, France; 5 Department of Food Science, Fu Jen Catholic University, New Taipei City 24205, Taiwan; 6 Biotechnology Program, Dr. Rammanohar Lohia Avadh University, Ayodhya 224001, India

**Keywords:** bioremediation, microbes, pollutants, environment, sustainable technologies

## Abstract

Environmental pollution brought on by xenobiotics and other related recalcitrant compounds have recently been identified as a major risk to both human health and the natural environment. Due to their toxicity and non-biodegradability, a wide range of pollutants, such as heavy metals, polychlorinated biphenyls, plastics, and various agrochemicals are present in the environment. Bioremediation is an effective cleaning technique for removing toxic waste from polluted environments that is gaining popularity. Various microorganisms, including aerobes and anaerobes, are used in bioremediation to treat contaminated sites. Microorganisms play a major role in bioremediation, given that it is a process in which hazardous wastes and pollutants are eliminated, degraded, detoxified, and immobilized. Pollutants are degraded and converted to less toxic forms, which is a primary goal of bioremediation. *Ex situ* or *in situ* bioremediation can be used, depending on a variety of factors, such as cost, pollutant types, and concentration. As a result, a suitable bioremediation method has been chosen. This review focuses on the most recent developments in bioremediation techniques, how microorganisms break down different pollutants, and what the future holds for bioremediation in order to reduce the amount of pollution in the world.

## 1. Introduction

Pollution of the environment, freshwater, and topsoil has evolved from global industrialization. Water quality has worsened as a result of human activity, such as due to mining and ultimate removal of toxic metal effluents from steel mills, battery companies, and electricity generation, posing major environmental concerns. Effluents like petroleum, polythenes, and trace metals harm the environment. Heavy metals are pollutants that exist in nature in the Earth’s crust and are difficult to decompose. They exist as ores in rocks and are recovered as minerals. High-level exposures can release heavy metals into the environment. Once in the environment, they remain toxic for much longer [1]. Many of these pollutants are mutagenic to both humans along with their surroundings. Absorbing heavy metals accumulates in the brain, liver, and kidney. Other effects on animals include cancer, nervous system damage, stunted growth, and even death [2]. Heavy metals in soils reduce food quality and quantity by inhibiting nutrient absorption, plant growth, and physiological metabolic processes. Metal-contaminated soils are being remedied using chemical, biological, and physical methods. However, physicochemical methods produce a lot of waste and pollution, so they are not valued [3]. Bioremediation is a cost-effective and practical solution for removing environmental contaminants [4]. Plant growth promotion, insect control, soil conservation, nutrient recycling, and pollutant reduction are all key functions of soil microorganisms [5]. Bioremediation has come a long way in terms of efficiency, cost, and social acceptability [6]. Bioremediation research has largely focused on bacterial processes, which have numerous applications. Archaea are known to play a role in bioremediation in many applications where bacteria are involved. Many hostile situations have degraded, requiring bioremediation. Microbes can also assist in the elimination of pollutants from hyperthermal, acidic, hypersaline, or basic industrial waste [7,8]. Recent research suggests that using more than one living organism will improve the efficiency and results, and allow for greater microbial diversity in bioremediation [8,9]. Many researchers employed bioremediation technology for the removal of organic and inorganic pollutants [10,11,12]. In a study, bioremediation technology was used for the treatment of various pollutants, including organophosphate pesticides such as chlorpyrifos, methyl parathion, and profenofos, by *Aspergillus sydowii*, and chloramphenicol by endophytic fungi, respectively [13,14]. In another study, *Cymbella* sp. has been shown to detoxify naproxen-polluted water with an efficiency of 97.1% [15].

A bioremediation approach requires the use of microbial enzymes to break down hydrocarbons into less harmful compounds. The widespread use of genetically-modified microorganisms that can also help to eliminate petroleum, naphthalene, toluene, benzene, and other xenobiotic chemicals is now being studied [16]. Several factors, such as temperature of the surrounding environment, aerobic or anaerobic conditions, and nutrient availability, all influence bioremediation for better outcomes. Emerging environmental pollutants, such as persistent organic compounds, heavy metals, toxins, and air pollutants that are of synthetic or natural origin, reach ecosystems mainly through anthropogenic activities and pose adverse threats to lifeforms like plants, animals, and humans [17]. One of the most economical and environmentally favorable biotechnological innovations is bioremediation. Waste management mainly relies on bioremediation. It can remove persistent organic pollutants, which are hard to breakdown and are thought to be heterologous biological substances. This review addresses the recent approaches and updated information of bioremediation strategies for eco-friendly detoxification and the effective degradation of various organic and inorganic contaminants to control environmental pollution.

## 2. Microorganisms Used in Bioremediation

Biological equilibrium is maintained in part by the contribution of microorganisms to nutritional chains. Bioremediation is the process of using bacteria, algae, fungi, and yeast to remove contaminated materials from the environment [18]. In the presence of hazardous compounds or any waste stream, microbes can grow at temperatures as low as −196 degrees Fahrenheit and as high as 1200 degrees Fahrenheit. The adaptability and biological systems of microbes make them an ideal choice for remediation [19]. Carbon is the most important nutrient for microorganisms. Microbes from a variety of environments were used to perform bioremediation. *Achromobacter*, *Alcaligenes*, *Xanthobacter*, *Arthrobacter*, *Pseudomonas*, *Bacillus*, *Mycobacterium*, *Corynebacterium*, *Flavobacterium*, *Nitrosomonas*, and other microorganisms [9] are examples of microbes.

### 2.1. Aerobic

Several microorganisms have the ability to bioremediate different types of environmental pollutants under aerobic conditions. *Bacillus*, *Pseudomonas*, *Sphingomonas*, *Flavobacterium*, *Nocardia*, *Rhodococcus*, and *Mycobacterium* are aerobic bacteria that can degrade a variety of complex organic compounds [20]. Pesticides, alkane hydrocarbons, and polyaromatic compounds have been shown to be degraded by these microbes. Several of these microorganisms make use of these contaminants as a source of carbon and energy [21]. In the aerobic bioremediation process, oxygen is the limiting factor for the growth of microorganisms.

### 2.2. Anaerobic

Amphibious bacteria that degrade and convert pollutants to fewer toxic forms are becoming increasingly popular for the bioremediation of polychlorinated biphenyls, chlorine compounds, and the chlorinated solvents, trichlorethylene and chloroform [22]. Several bacteria, such as *Pseudomonas*, *Aeromonas,* and sulfate-reducing bacteria, have been used in the bioremediation process under anaerobic conditions. Garg and Tripathi [23] reported microbial discoloration of azo dyes under different environmental situations. Azo dyes can decompose anaerobically through reduction reactions using electrons produced by the oxidation of the organic substrate(s). Due to such controlled dye decolorization events, microbe electrochemical properties would have a major impact on the effectiveness of color removal. Dyes were anaerobically decolored for industrial activities to progressively acquire such time-variant decolorized-metabolites (DMs). However, external augmentation of DMs gathered under certain conditions was carried out for improved research so that a precise system can be used [24].

## 3. Factors Affecting Microbial Bioremediation

Bioremediation is the process of using microorganisms such as bacteria, algae, fungi, and plants to break down, change, remove, immobilize, or detoxify various physical and chemical pollutants in the environment. Microorganisms’ enzymatic metabolic pathways speed up biochemical reactions that break down pollutants [25,26]. In order for microorganisms to combat pollutants, they must come into contact with compounds that provide them with the energy and nutrients they need to multiply. There are several factors such as physical, chemical, biological, soil-type, carbon and nitrogen source, type of microorganisms—i.e., single or consortium—and others that affect the process of bioremediation [27]. Microbial consortiums often have both multifunctionality and resistance because different species work together to use all substrates in the best way possible, thereby increasing the bioremediation efficiency compared to single microorganism [28]. In a study, carbon is one of the most important nutrients that help *in situ* bioremediation by increasing the metabolic activity of natural microbial communities and speeding up the bioremediation process to break down existing pollutants. Bioremediation may use organic carbon more than any other additive. In an anaerobic environment, many microorganisms can ferment organic carbon and make hydrogen gas [29]. In a study, bioremediation was found to be significantly affected by soil types, and the removal efficiency of pollutants varied in sandy soil and clay soil, respectively [30]. For bioremediation to be a success, it must be able to access existing microorganisms as well as the environment’s physicochemical characteristics (Table 1). The microbial population responsible for degrading pollutants, the accessibility of contaminants, and the following factors are taken into consideration.

## 4. Principle of Bioremediation

When organic wastes are biologically degraded under controlled conditions, “bioremediation” is the term used to describe this process. Using bioremediation, harmful substances can be degraded or detoxified by providing the organisms with the nutrients and other chemicals they need to function optimally. Enzymes play a critical role in every stage of the metabolic process [24,43]. It is part of the family of oxidoreductases, lyases, transferases, and hydrolases. Non-specific and specific substrate affinities allow many enzymes to degrade a wide range of substrates. There must be enzymatic action on the pollutants in order for bioremediation to be successful. In order to speed up microbial growth and degradation, environmental parameters must often be manipulated during bioremediation [38,43]. This is because bioremediation only works when the environment is right for microbes to grow and move around.

Living organisms and fertilizers can aid in the process of bioremediation, which occurs naturally and is encouraged. Biodegradation is a key component of bioremediation technology. It’s the process of converting harmful organic pollutants like carbon dioxide and water into non-toxic or naturally-occurring inorganic compounds that are safe for use by humans, plants, animals, and aquatic life [44].

## 5. Types of Bioremediations

**Figure 1:**
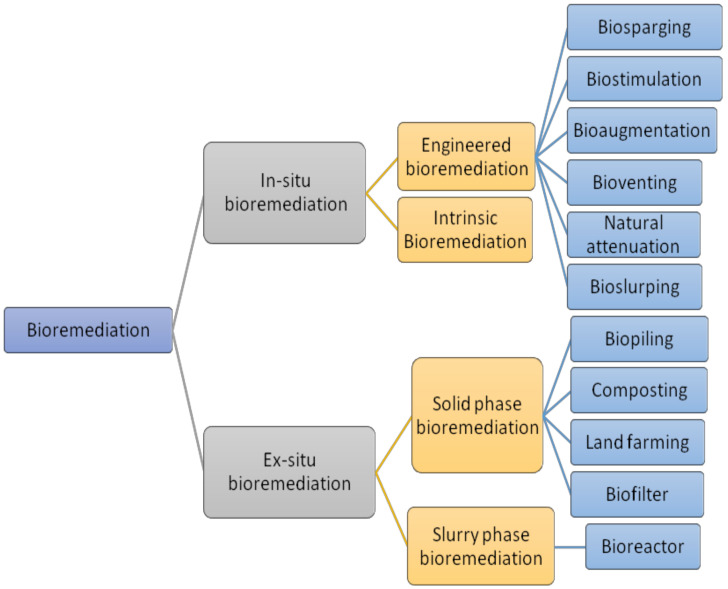
Diverse bioremediation techniques.

Bioremediation can be used in a plethora of ways, and some of the most commonly used methods are presented here (Figure 1).

### 5.1. Biopile

In bioremediation, aeration and nutrient supplementation are used to enhance microbial metabolic activities in the piled-up polluted soil above ground. Aeration, nutrients, irrigation, leachate collection, and treatment bed systems are all included in this procedure. When it comes to *ex situ* biodegradation, this method is becoming increasingly popular because of its cost-effectiveness and useful features, such as pH and nutrient control. Using the biopile to clean up polluted cold environments and treat low-molecular-weight volatile pollutants is an option [15,45]. The biopile’s adaptability allows for a reduction in remediation time by increasing microbial activity and contaminant availability while also increasing biodegradation rate. When warm air is introduced into the biopile system to provide air and heat simultaneously, bioremediation is improved. The biopile’s remediation process has been helped by the addition of bulking agents like straw, sawdust, or wood chips. To replenish the air supply to polluted piled soil in biopiles, *ex situ* bioremediation techniques such as land farming, biosparging, and bioventing can be applied [46]. However, these techniques are expensive to implement and require a power supply at remote locations. Bioremediation may be slowed down by extreme air temperatures that dry soil and make it more likely to be vaporized than to be broken down by living organisms [47]. Bio-available organic carbon (BOC) plays an important role in bioremediation through the biopile method. Petroleum contaminated soil has been bioremediated using mesophillic conditions (30 °C–40 °C) and a low aeration rate for the removal of total petroleum hydrocarbon (TPH) using *alpha*, *beta*, and *gamma proteobacteria* [48]. Biopile systems have also been utilized for treating the diesel contaminated soil of the sub-Antarctic region. A total of 93% of the total petroleum hydrocarbon (TPH) was removed using the biopile system within one year [49].

### 5.2. Windrows

Windrows boosts bioremediation by enhancing the biodegradation processes of native and transitory hydrocarbon plastic found in the contaminated soils when spinning the heaped contaminated soils. The aeration, mineralization, and biotransformation of toxic soil can be performed through acclimation, biological treatment, and mineralization [50], can speed up bioremediation. The biopile approach can remove more hydrocarbons from soil than windrow treatment [15,51], which was more efficient in terms of hydrocarbon removal. The periodic rotation connected with windrow remediation is not a better selection approach for the bioremediation of soil affected by harmful volatile chemicals. Windrow treatment is a source of greenhouse gas (CH_4_) due to the anaerobic system generated inside the heaped contaminated soil [52]. The windrow method of has been applied for the bioremediation of the Gurugram–Faridabad dumpsite in Bandhwari, India by forming terraces and windrows and utilizing bio-culture, and the results showed a decrease in the garbage [53].

### 5.3. Land Farming

Land farming is the most significant and simple bioremediation method because of its low operating costs and lack of specialized equipment [54]. *Ex situ* bioremediation is the most common method, but it can also occur with *in situ* bioremediation. The reason for this is the location of the treatment. It is common practice in land farming to remove and till polluted soils on a regular basis, and the location of treatment dictates the type of bioremediation. On-site treatment is classified as *in situ*, whereas *ex situ* bioremediation approaches are used for the treatment of the contaminated soil [55]. Extracted contaminated soils are usually placed on a permanent layer of substrate well above Earth’s surface to permit native microorganisms to aerobically degrade contaminants [56]. Land bioremediation of polluted soil using land farming bioremediation technology is a reasonably simple process that takes little capital, has little ecological footprint, and uses very little energy [57].

### 5.4. Bioreactor

Following a series of biological reactions, bioreactors transform raw materials into specific products. Bioremediation thrives in a bioreactor, which provides the ideal conditions for growth [58]. The remediation samples are placed in a bioreactor. There are a number of advantages to using a bioreactor to treat contaminated soil as opposed to *ex situ* bioremediation methods. An efficient bioremediation process based on bioreactors that can precisely regulate pH, agitation, temperature, aeration, substrate concentration, and inoculum concentration significantly reduce the time required for bioremediation [59]. Biological reactions can take place when the bioreactor can be controlled and manipulated. Given their adaptability, bioreactor designs are able to maximize microbial degradation while abiotic losses are kept to a minimum.

#### *In Situ* Bioremediation Techniques

These methods entail cleaning up polluted substances right where they were created. It does not necessitate any digging or disturbance of the surrounding soil. These techniques ought to be more cost-effective in comparison to the *ex situ* bioremediation techniques. Bioventing, phytoremediation, and biosparging are examples of *in situ* bioremediation techniques that can be improved, while intrinsic bioremediation and natural attenuation are examples of *in situ* bioremediation techniques that cannot be improved [60]. *In situ* bioremediation approaches have effectively treated chlorine, paints, toxic metals, and hydrocarbon-contaminated areas [61]. The practice of *in situ* bioremediation can be categorized into two distinct types: intrinsic and engineered.

(a) Intrinsic *in situ* bioremediation:

Natural reduction is another term for *in situ* bioremediation. Intrinsic bioremediation utilizes polluted sites in a non-invasive manner (human intervention) [62]. The goal of this procedure is to stimulate an already existing microbial population. The biodegradation of polluting constituents, including those that are recalcitrant, is based on aerobic and anaerobic processes in microorganisms. It costs less because there isn’t a lot of force behind this technique [63]. Intrinsic *in situ* bioremediation can be performed using anaerobic reductive dechlorination, aerobic treatment, amendment delivery, biosparging, and bioslurping [64]. Using a stimulation–optimization approach that is powered by machine learning and particle swarm optimization (ELM–PSO) techniques, *in situ* bioremediation has been used as a method for the biological treatment of clogged groundwater [65]. This technique was implemented through the use of *in situ* bioremediation. This results in cheaper technology for the pumping system and requires less capital for the whole process. The concentration of contaminants was reduced from 40 ppm to 5 ppm (within permissible range) in 3 years using *in situ* bioremediation. *In situ* remediation has also been explored for the decontamination of Cr (VI) found in shallow unsaturated soil. Microorganisms possess the capability to survive under high concentrations of Cr (VI) in the soil and their sub-cellular machinery was utilized to interact with heavy metals. Microbial inoculants can be utilized for the *in situ* treatment of heavy metals [66]. Cr (VI) interacts with Fe (II) ions also through the redox reactions, and the release of iron in soluble forms promotes the reductive reactions [67].

(b) Engineered *in-situ* bioremediation

In the second method, a specific microorganism is brought into the area of contamination to clean it up. *In situ* bioremediation is a technique that employs microorganisms that have undergone genetic engineering in order to hasten the decomposition process. This is accomplished by enhancing the physicochemical conditions that foster the growth of microorganisms [68].

### 5.5. Bioventing

Bioventing is a technique that uses controlled airflow to increase the activity of indigenous microbes for bioremediation by delivering oxygen to the unsaturated zone. The bioremediation process is aided by the addition of nutrients and moisture during the bioventing process. This will lead to the microbial transformation of pollutants into harmless substances. Other *in situ* bioremediation methods have flocked to this one in recent years [69]. Bioventing is a technique that helps in stimulating the indigenous microflora through ample amounts of aeration to enhance the biodegradation ability of the various microbes and promote decontamination of the heavy metal pollutants by precipitation [70].

### 5.6. Bioslurping

A direct oxygen supply and stimulation of contaminant biodegradation are used in conjunction with vacuum-assisted pumping, bioventing, and soil vapour extraction (SVE) in order to reach soil and groundwater levels for restoration [71]. This approach can be used to recover unsaturated and saturated zones as well as light non-aqueous phase liquids (LNAPLs). This technology can be used to remediate soils contaminated with flammable and moderately-flammable organic substances. Liquid is drawn from the free product layer by means of a “slurp” that spreads into the layer. LNAPLs are lifted to the surface by the pumping machine, where they are separated from the surrounding air and water [72]. To reduce microbial activity, soil moisture is used in this technique to reduce air permeability and oxygen transfer rate. Given that it uses less groundwater, this method saves money on storage, disposal, and treatment, even though it’s not ideal for remediation in low-permeable soils. Bioslurping requires 25 feet of digging below the ground surface and then the contaminants floating on the water can be removed. It combines both the approaches of bioventing, which utilize aerobic bioremediation of contaminated soil *in situ.* Free product is recovered by a vacuum-enhanced system that utilizes LNAPLs from the capillary fringe [73]. Free product is “slurped” up the bioslurping tube into a trap or oil–water separator for further treatment after the bioslurping tube is vacuumed. When the LNAPL is removed, the height of the LNAPL drops, which encourages the flow of LNAPL from distant locations into the bioslurping well. The bioslurping tube starts to remove vapours from the unsaturated zone when the fluid level in the bioslurping well decreases as a result of the vacuum extraction of LNAPL. This vapour extraction encourages soil gas movement, which in turn boosts aerobic biodegradation and aeration [74].

### 5.7. Biosparging

Air is introduced into the soil’s core, just like bioventing, to encourage microbiological activity, which in turn removes pollutants from polluted sites. As an alternative to conventional biodegradation methods, bioventing involves injecting air into a saturated zone in order to encourage the movement of flammable organic chemicals upward to an unsaturated zone nearby [75]. The success of biosparging is dependent on soil porosity and contaminant biodegradability. When it comes to bioventing and soil vapour extraction (SVE), *in situ* air sparging (IAS) uses high air-flow rates to volatilize contaminants, while biosparging encourages microbial degradation [76]. It is common practice to use biosparging to remove diesel and kerosene from water supplies. In order to hasten the biodegradation processes, oxygen is supplied into microorganisms during enhanced bioremediation [77]. The removal of organic pollutants (BTEX) can be accomplished using a variety of technologies, including adsorption, microbial degradation, biosparging, PRBs, and the use of modified or synthesized zeolites. However, there aren’t many investigations on readily available, inexpensive materials like natural zeolite for BTEX adsorption [78].

### 5.8. Phytoremediation

Contaminated soils can be cleaned up using phytoremediation. In contaminated areas, this method uses plant interactions at the physical, biological, chemical, biochemical, and microbiological levels to reduce pollutant toxicity. Depending on the quantity and form of the pollutant, phytoremediation employs a variety of processes [79]. Extraction, sequestration, and transformation are common methods for removing pollutants like heavy metals. When using plants like willow or alfalfa, the decay, immobilization, rhizoremediation, and evaporation of organic contaminants such as oils and chloro-compounds is feasible [80]. Tap root system or fibrous root system, penetration, toxicity levels, adaptability to the harsh environmental conditions of the contaminants, plant annual growth, supervision, and, notably, the time needed to reach standard of cleanliness are all important factors in plants that serve as phytoremediators. The plant must also be disease and insect resistant [81]. An important part of phytoremediation is removing pollutants from the roots and shoots. The movement of water and nutrients is also dependent on transpiration and partitioning [82]. When it comes to contaminants and plant nature, it is possible to alter this process. Phytoremediation can be accomplished with the help of the majority of the plants present at a polluted site. In polluted environments, native plants can be bioaugmented by natural or anthropogenic plants, or a combination of both. Phytomining, the process of extracting precious metals from polluted sites with plants, is one of them [83].

Numerous plants (over 300) are better candidates for phytoremediation because they ideally absorb Cu, Zn, and Ni. Phytostabilization, sometimes referred to as *in situ* inactivation or immobilisation of heavy metals, reduces their bioavailability and prevents their off-site transfer. At the plant roots, it absorbs metals and restores them. Several species, notably *Acanthus ilicifolius* and *Virola surinamensis*, are capable of Cd photostability. *Cinnamomum camphora*, *Osmanthus fragrans*, *Euonymus japonicus*, *Ligustrum vicaryi*, and *Loropetalum chinense* are five decorative plants chosen for their capacity to phytostabilize Cd [84]. Water from various places that has been contaminated with metal can be successfully treated using bacterially-aided phytoremediation. The phytoremediation method of metal reduction in wastewater utilising plants can be used by coalitions of growth-promoting rhizobacteria, degrading bacteria, as well as endophytic bacteria [85]. There are a few limitations to bioremediation techniques, as presented in Table 2.

## 6. Bioremediation of Various Pollutants

### 6.1. Bioremediation for Organic Pollutants

Organic compounds (OCs) such as biocides and flame retardants have been widely used and are now considered a threat to nearly all forms of life on the planet because of the widespread and massive use of these chemicals in the environment. Most OCs, such as polychlorinated biphenyls (PCBs), polybrominated biphenyl ethers (PBEs), and polycyclic aromatic hydrocarbons (PAHs), can be degraded in the environment by microbes. Biodegradation is the process by which microbes break down organic compounds into less toxic or entirely non-toxic residues [91]. In order to obtain organic carbons and energy, the microbes consume the organic substrate. Isolated from other microbes, an individual microbial species usually does not degrade any organic substrate [92] and does well in a community. As a result of community microbe interactions, resistance, chemical-degrading ability, and tolerance are all conferred by the exchange of genetic information among microbial species. Many OC-degrading microorganisms are misidentified due to a lack of internationally agreed-upon methods and protocols for microbial identification [93]. This underlines the significance of studies into microbial consortiums using metagenomics tools and conventional genetic engineering protocols. Bacteria and other microorganisms have the ability to degrade a wide range of organic compounds, depending on the chromosomal genes, as well as the extracellular enzymatic activity (in the case of bacteria) (fungal degradation process). The varying environmental conditions that affect the microbe growth pattern further complicate these processes [94]. 

A successfully bioengineered microbe requires the identification of the relevant species and strains for each substrate. A viable alternative to the recombinant degradation of resistant organic compounds is biodegradation by microbes using readily-available organic carbon and energy sources in the surrounding environment. Microbes use the fluctuation in chemical gradients in their environment to determine the most favourable conditions for growth. This allows them to thrive in an optimal environment [95]. Microbial consortia and microbial fuel cells (MFCs and bioreactors) are two new developments in microbiological bioremediation that are being used to degrade recalcitrant organic compounds. Toxic organics can be remedied more effectively using fungi rather than bacteria because the latter cannot grow at high concentrations of toxic organics [96]. For example, the enzymes, laccase (LAC), lignin peroxidase (Lip), and manganese decarboxylase (MDA), are active in the metabolism of lignocellulosic compounds by the white rot fungus *Phanerochaete chrysosporium* [97].

### 6.2. Bioremediation for Inorganic Pollutants

Toxic heavy metals and their compounds resulting from mining, power plants, metallurgy, and chemical manufacturing processes are among the most common inorganic contaminants [98]. One of the main concerns of environmentalists is toxic elemental pollution because the disposal of toxic metals to soils and waters on or below the surface causes unacceptable health risks [99]. Microbes cannot degrade metal ions; it is essential to know that they are only capable of changing the oxidation states of the metals to stabilize them [100]. They can metabolize and detoxify metals like any other nutrient in the cells. Several microorganisms have been reported for the bioremediation of organic and inorganic pollutants (Table 3). Microbes that release chelating agents and acids, as well as those that alter physicochemical properties such as redox potential in their environment can cause significant changes in the environment by increasing the bioavailability of metal ions [101]. Physical adsorption, biosorption, and ion complexation are the first steps in the interaction between metals and microbial cells [102]. Enzymes for oxidation, methylation, reduction, precipitation, and dealkylation are involved in the biochemical transformation of metal ions by microorganisms. The adaptability of microbes to heavy metals, such as iron, zinc, chrome, magnesium, mercury, and barium in textile waste, was demonstrated in the multidrug-resistant *Pseudomonas aeruginosa* T-3 isolate from tannery effluent [67,83]. This shows that microbes can adapt to changing environmental conditions. A plasmid-encoded copper and cadmium metal resistance gene in the bacteria, *Pseudomonas putida* PhCN, has also been discovered [103]. Plasmid-encoded biochemical information and genetic engineering techniques were used to create recombinant *Escherichia coli* that expresses the metallothionein gene (*Neurospora crasa*) for Cd uptake, resulting in significantly faster Cd uptake than the donor microbe [104]. A poly-histidyl peptide was introduced into *Staphylococcus xylosus* and *Staphylococcus carnosus* that encoded genes that allowed these microbes to bind nickel [105].

## 7. Recent Advancement and Challenges in Bioremediation

### 7.1. Bioinformatics Approaches in Bioremediation

When it comes to waste management, bioremediation is a useful technique that can be used to remove waste from contaminated areas and sites. It is particularly concerned with the utilization of organisms to consume or neutralize pollutants [20]. Using data from various biological databases, such as databases of chemical structure and composition, RNA/protein expression, organic compounds, catalytic enzymes, microbial degradation pathways, and comparative genomics to interpret the underlying degradation mechanism carried out by a particular organism for a specific pollutant is the goal of bioremediation [133]. A variety of bioinformatics tools are used to interpret all of these sources in order to study bioremediation in order to develop more effective environmental cleaning technology. There has been a scarcity of data on the factors that control the growth and metabolism of microbes with bioremediation potential, which has resulted in a limited number of bioremediation applications [134]. These microorganisms with bioremediation capabilities have been profiled and their mineralization pathways and mechanisms have been mapped out using bioinformatics [135]. The use of proteomic approaches such as two-dimensional polyacrylamide gel electrophoresis, microarrays, and mass spectrometry is also critical in the investigation of bioremediation methods and technologies. It significantly improves the structural characterization of microbial proteins that have contaminant-degradable properties, according to the researchers [135]. The structural characterization of microbial proteins capable of degrading contaminants has greatly improved. Research in this field crosses the boundaries between computer science and biology. For example, computers are used to store, manipulate, and retrieve information linked to the DNA, RNA, and proteins of the genome [133,135].

#### 7.1.1. Bioremediation Tools Based on Omics

Bioremediation studies can benefit from the use of genomics, transcriptomics, metabolomics, and proteomics. Given its ability to correlate DNA sequences with the abundance of metabolites, proteins, and mRNA, this technology aids in the *in situ* bioremediation process’s evaluation [136,137].

#### 7.1.2. Genomics

There is a new field in genomics for the study of bioremediation microbes. This strategy is based on microbes’ ability to fully analyze their genetic information within the cell. Bioremediation uses a wide variety of microorganisms [138]. To better understand the biodegradation process, genomic tools such as PCR, analysis of isotope distribution, DNA hybridization, molecular connectivity, metabolic footprinting, and metabolic engineering are used. For genotypic fingerprinting, a variety of PCR-based techniques are available, including amplified fragment length polymorphisms (AFLP), amplified ribosomal DNA restriction analysis (ARDRA), automated ribosomal intergenic spacer analysis (ARISA), terminal-restriction fragment length polymorphism (T-RFLP), randomly amplified polymorphic DNA analysis (RAPD), single strand conformation polymorphism (SSCP), and length heterogeneity [139]. When it comes to studying soil microbial communities, RAPD can be utilized for assessing inherently related bacterial species, constructing functional structural models, and generating genetic fingerprints [140]. In microbial communities, LH-PCR may be used to detect natural length variations of various SSU rRNA genes. Multiple taxonomic groups of microbes can be profiled simultaneously using T-RFLP [141]. Research into how soil microbes interact with natural factors can also make use of a combination of molecular tools, such as genetic fingerprinting, microradiography, FISH, stable isotope probing, and quantitative PCR. A PCR-based quantitative analysis of soil microbial communities can be used to determine the abundance and appearance of taxonomic and operational gene markers in the soil. Techniques for analysing a person’s DNA use amplified PCR products as a starting point for the direct analysis of specific molecular biomarker genes [142]. In order to better understand the relationship between diverse microbial communities, cluster-assisted analysis, which compares fingerprints from different samples, could be used.

#### 7.1.3. Transcriptomics and Metatranscriptomics

The transcriptome is a crucial link between cellular phenotype, interactome, genome, and proteome because it represents the set of genes that are being transcribed at a specific time and condition. The ability to control gene expression is critical to adapting to changes in the environment and thus ensuring survival. Transcriptomics provides a comprehensive view of this process across the entire human genome. In transcriptomics, DNA microarray analysis is a powerful tool for determining mRNA expression levels [143]. To perform a transcriptomic analysis, one must first isolate and enrich the total mRNA, then synthesize cDNA, and then sequence the cDNA transcriptome. Using a DNA microarray as a transcriptomics tool, almost every gene in an organism’s mRNA expression can be examined and studied [144]. The study of transcriptional mRNA profiles, also known as transcriptomics or metatranscriptomics, is critical for gaining functional insights into the activities of environmental microbial communities [145]. Syntrophism between microbes and complementary metabolic pathways can be discovered using metagenomics and genome binning as well as metatranscriptomics during the entire biodegradation process [146]. Metatranscriptomics is a way to look at gene expression that can be used by researchers [147].

#### 7.1.4. Proteomics and Metabolomics

In contrast to metabolomics, which focuses on the total metabolites produced by an organism in a given period of time or environment, proteomics focuses on the total proteins expressed in a cell at a given location and time [148]. The analysis of protein abundance and changes in composition, as well as the identification of key microbe-related proteins, has been accomplished using proteomics [149]. In comparison to genomics, the functional analysis of microbial communities is more useful and holds more promise. There are two primary ways in which metabolomics studies can be used to analyze biological systems. It is not necessary to have any prior knowledge of the metabolic pathways of the biological system in order to conduct the first type of study. By employing this strategy, there are numerous metabolites in the sample that can be identified and recovered, which generates enormous amounts of data that can be used to establish the interconnectedness of various samples in metabolic pathways. Another option is to conduct a targeted study to identify specific metabolic pathways or metabolites based on prior research [150]. Metabolite profiling, foot printing, and target analysis are just some of the many tools in the toolbox of microbial metabolomics that can be used to identify and quantify the myriad of cellular byproducts present in living organisms [151]. Data from both the proteome and metabolome will be useful for cell-free bioremediation.

### 7.2. Bioremediation Using Nanotechnological Methods

A nanometer is the smallest unit of measurement used in nanotechnology. Many toxic substances can be removed with their help because of their unique abilities against various recalcitrant contaminants. Technology such as water treatment has been given a new perspective by nanotechnology. Techniques that are good for the environment can now be categorized as nanofiltration [151].

#### 7.2.1. Microbe and Nanotechnology

When using effective microbes (EM) technology, wastewater can be treated with effective microbes, and the water can then be used for irrigation [152]. For water purification, nanotechnology and EM technology can be helpful. Innumerable and all-pervasive environmental issues arise from the presence of recalcitrant organic pollutants like polycyclic aromatic hydrocarbons (PAHs) with multiple benzene rings. Polycyclic aromatic hydrocarbons (PAHs) are mutagenic and non-biodegradable [153]. In a study, Ramos et al. [154] synthesized silver nanoparticles using whole cells of the fungi *Trichoderma* spp. for its application.

#### 7.2.2. Engineered Polymeric Nanoparticles for Hydrophobic Contaminant Bioremediation

Soil sorption of organic pollutants, such as petroleum hydrocarbons and PAHs, reduces their solubility and mobility, which in turn reduces their environmental impact. The phenanthrene solubility and phenanthrene release from contaminated aquifer material are both improved by polymeric nano-network particles [155]. Precursor chains of poly-(ethylene) glycol-modified urethane acrylate (PMUA) are used to create polymeric nanoparticles. PMUA nanoparticles are designed to maintain their properties in the presence of a diverse range of bacterial populations [156].

### 7.3. Genetic and Metabolic Engineering

“Gene editing” refers to scientific technical developments that enable rational genetically-created fragments at genome level to provide exact addition, deletion, or substitution of pieces of DNA molecules. Transcription activators are utilized in a variety of gene editing methods, including TALENs, ZFNs, and CRISPRs, which are widely used in research. CRISPR-Cas has been dubbed the most efficient and straightforward gene editing tool [157]. A DNA-binding element in TALEN is complementary to the sequence of the host DNA. When TALEN attaches to DNA and exposes sticky ends for stabilization, it creates double-stranded breaks (DSBs). ZFNs also have a DNA-binding domain made up of 30 amino acids. At the target location of the host DNA, the Fok1 cleavage domain causes DSBs. A novel perspective on composite endonuclease comprising TALENs and ZFN nucleases was required to solve molecular problems [158,159]. Two of the CRISPR-Cas system’s unique properties are sequence similarity complementarity and simultaneous gene editing [160,161]. The bacteria, *Streptococcus pyogenes,* provides this unique ability as a sort of virus resistance. In the CRISPR-Cas system, guide RNA connects crisper-derived RNA (crRNA) and trans-acting antisense RNA (trcRNA). The Cas9 enzyme is able to carry out the requisite DSB when gRNA recognizes the target DNA sequence. These gene editing tools’ knock-in and knock-out effects are being analyzed for usage in bioremediation investigations [161]. In model organisms like *Pseudomonas* and *Escherichia coli*, the CRISPR-Cas system has been widely accepted by researchers [138]. In non-model species (such as *Rhodococcus ruber* TH, *Achromobacter* sp. HZ01, and *Comamonas testosteroni*), the area of bioremediation is also exploring new insights into CRISPR toolkits and the synthesis of gRNA for the production of remediation-specific genes [162].

Pollutant-tolerant bacteria are the greatest choices for genetic manipulation and biochemical pathways since they are accustomed to tolerating and storing a variety of toxic, refractory, and non-degradable xenobiotic compounds under harsh circumstances. Furthermore, recognizing biochemical functions is critical for analyzing microbiological bioremediation, such as the bioremediation of harmful pollutants through the production of haloalkane dehalogenases and the disposal of pyrethrins from land through the anaerobic decomposition pathway of fenpropathrin studied in *Bacillus* sp. DG-02 [163]. The bioremediation process can be improved by metabolic engineering, which alters the existing pathway. The likelihood of obtaining recombinant enzymes increases significantly when using a genetic approach. Some extracellular enzymes have been found to play a role in enzymatic bioremediation, according to some studies. PAHs are degraded by LiPs ((lignin peroxidase) from *P. chrysosporium* that encode hemoproteins [164]. Even though contaminants can be consumed by microbes as substrates or intermediates in biological pathways, incomplete or partial degradation leads to simpler, non-toxic degradable compounds [136]. For example, LiP can degrade benzopyrene into three quinine compounds, namely 1,6-quinol, 3,6-quinine, and 6,12-quinine [165]. MnP (Mn (II) peroxidase) can also oxidise organic compounds in the presence of MnP (Manganese peroxidase) [166]. As well, laccase, glutathione S transferase, and cytochrome P_450_ are involved in the biodegradation of recalcitrant compounds [167]. It has been shown that the immobilization of enzymes significantly increases enzyme stability, activity, and stability. Enzymatic bioremediation is a simple, environmentally-friendly, and fast method for removing and degrading persistent xenobiotic compounds by microorganisms [134,145]. Enzyme-producing microorganisms have been isolated and characterized with the limitation of low productivity. Insecticides’ main ingredients, organophosphates (OP) and organochlorines (OC), are found in agricultural soil and run-off into waterways.

Genetically-engineered microorganisms have demonstrated successful bioremediation of hexachlorocyclohexane and methyl parathion [135,146]. Genetically-modified *P. putida* KT2440 was used in organophosphate and pyrethroid bioremediation experiments [168]. The degradation and catabolism of a variety of persistent compounds has been documented since the advent of metabolic engineering. *Sphingobium japonicum* and *Pseudomonas* sp. WBC-3 showed bioremediation of methyl parathion and -hexachlorocyclohexane degradation pathways [169]. When three enzymes from two different microorganisms are combined in *E. coli*, a persistent fumigant called 1-, 2-, 3-trichloropropane is released into the environment via heterologous catabolism [137,148]. To do this, microbes can be used to turn persistent compounds into minerals [49].

### 7.4. Designing the Synthetic Microbial Communities

Synthetic biology advancements have had a significant impact on environmental issues in recent years. Toxic compounds, pesticides, and xenobiotics can be removed from the environment by using genetically-modified organisms (GMOs). Natural microbial communities must be understood in order to create a synthetic one [170]. Identifying which species are participating in bioremediation is difficult in a natural community. Through the use of a synthetic microbial community, the development of an artificial microbiome with functionally specific species is possible. Model systems for studying functional and structural characteristics can be found in these communities. Synthetic communities were formed by the co-culturing of two distinct microorganisms under precisely defined conditions, which were based on their interactions and functions [171]. The community’s dynamics and structure are determined by these variables; it is based on the discovery of bacterial processes and behaviors. Metabolism drives these patterns of microbial interaction, which in turn facilitates communication within communities [172]. Interactions between two microbial populations are social in nature (such as mutualism, competition, and cooperation). Cooperation is said to be a key factor in community structure and operation. Cooperation in community dynamics is influenced by the creation of synthetic communities [173] and it was found that modifying environmental conditions, such as deleting genes, could be used to engineer cooperation between two microbial strains. In addition to this, the synthetic community’s engineered microbial species have been examined for other patterns of interaction. Bioremediation strategies frequently make use of this type of engineered interaction [174]. It is possible to sustain the existence of microorganisms in a large population by using synthetic biology.

## 8. Advantages and Disadvantages

**Figure 2:**
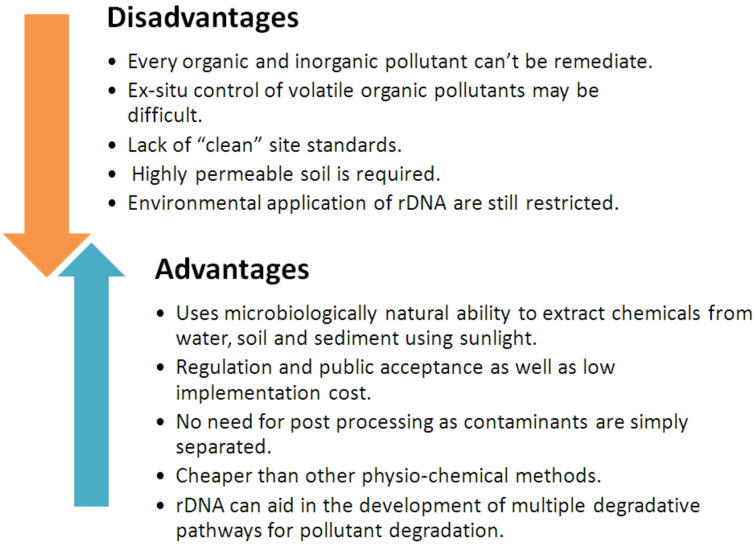
Advantages and disadvantages of bioremediation.

Due to the harm that pollutants exert on both humans and other living things, environmental pollution is a serious public health problem. The complete elimination of contaminants via chemical and physical methods of remediation is costly [175]. Additionally, both approaches may result in increased pollution and site disruption, which could have a detrimental effect on nearby humans and other biota. As a result, remediation techniques using chemicals and physical means are not regarded as eco-sustainable. Contrary to these techniques, bioremediation is the suggested solution to remove various persistent contaminants by relying on biological processes (mediated by various types of living organisms). However, all bioremediation techniques have their own advantages and disadvantages (Figure 2) because they have their own specific applications [176,177].

## 9. Future Perspectives and Conclusions

Omics has gained prominence in the field of microbial remediation of the pulp and paper industry, textile industry, food industry, dairy industry, wood industry, fisheries, water and soil treatment industry, solid waste remediation, heavy metal pollution remediation, and hydrocarbon remediation. In order to better understand degradative pathways, bioremediation data must be mined, and new algorithms can be used to fit these data into simulation and numerical modeling with ease along with data assemblage, repositioning, exploration, and transmission, which necessitate standard protocols. Bioremediation processes may be better understood if new biomarkers are studied. Combining all the omics data with genetically-engineered tools could provide a comprehensive picture of the microbial remediation process. The role of phytoremediation in reducing environmental pollution can also be studied. The phytoremediation process has a number of advantages over other remediation strategies, including lower costs, greater public acceptance, and increased pollution degradation capacity [178]. Groundwater and air pollution, along with toxic waste generation as a by-product of semiconductor manufacturing, are problems for the environment; some examples include glycol ethers, hydrochloric acid (HCl), xylene, hydrogen fluoride (HF), and methanol [179]. In the case of pharmaceuticals that are designed to be long-lasting or even non-degradable, they pose a unique threat to the environment. The pharmaceutical pollutants are environmentally persistent substances. Trace amounts of pharmaceutical ingredients, such as birth control pills, anti-epileptics, pain relievers, and antidepressant medications, are found in many urban and rural sources of groundwater [180]. While operating, solar power generation facilities produce less greenhouse gas emissions, including air pollutants such as carbon monoxide, volatile organic compounds, nitrogen oxides, and carbon dioxide, than conventional fossil fuel-based power generation facilities [181]. Genetically-engineered plants can be able to bioremediate specific pollutants through discovered metabolic processes, enzymes, genes, or operons [182]. Although genomics, metabolomics, and proteomics in bioremediation aid in the exploration of possible solutions to specific pollutants, identifying and comparing gene and protein sequences that are effective at removing contaminants is the next step in bioremediation research. GMOs can clean up a wide range of waste effluents and polluted land [183]. When used in conjunction with other physical and chemical methods, bioremediation can provide a comprehensive approach toward removing pollution from the environment. Since it appears to be a long-term solution, there is a need for additional research in this area.

## Figures and Tables

**Table 1:** Critical factors for microbial bioremediation.

Factors	Remarks	References
Biological factors	Soil microorganisms compete for carbon sources, or bacteriophages and protozoa prey on each other, all of which can affect organic compound degradation. Derivatization rates are influenced by contaminants and catalyst levels. Expressed enzymes can speed up or slow contaminant degradation. Enzymes must also be involved in contaminant metabolism to have an affinity for the contaminant and availability. The major biological factors: interaction (competition, predation, and succession), population size, and composition.	[31,32]
Oxygen	Biodegradation rates can be improved by using organisms that don’t require oxygen. Anaerobic decomposition occurs as most living organisms need oxygen to survive. In most cases, hydrocarbon metabolism can be boosted by the addition of oxygen.	[33]
Moisture content	Microorganisms require a sufficient amount of water to achieve their growth goals. When the soil is too wet, the biodegradation agents don’t work as well.	[34]
Nutrients	Nutrients can influence microbial growth and reproduction, as well as biodegradation rate and effectiveness. Optimizing the bacterial C:N:P ratio can improve biodegradation efficiency, especially when essential nutrients like N and P are supplied. Carbon, phosphorous, and nitrogen are just a few of the nutrients microorganisms need to survive. In low concentrations, hydrocarbon degradation is also limited. Adding nutrients to cold environments can increase microorganisms’ metabolic activity and thus the biodegradation rate. Aquatic biodegradation is limited by nutrient availability. Oil-eating microbes require nutrients to thrive. These essential nutrients are found in small amounts in nature.	[35,36]
Temperature	The most important physical factor influencing microorganism survival and hydrocarbon composition is temperature. In cold climates like the Arctic, natural oil degradation is slow, putting more pressure on microbes to clean up spilled oil. Here, the sub-zero water freezes the microbial transport channels, rendering them unable to perform their metabolic functions.Temperature affects the metabolic turnover of enzymes involved in degradation. Also, each compound’s degradation requires a specific temperature. Temperature affects microbial physiological properties and thus speeds up or slows down bioremediation. Increased microbial activity occurs at higher temperatures. It started to drop suddenly as the temperature increased or decreased, and theneventually stopped.	[37,38]
pH	A compound’s acidity, alkalinity, and basicity affect microbial metabolism and the removal process. Microbial growth can be predicted by the soil’s pH. Even minor pH shifts have a significant impact on metabolic processes.	[39]
Sitecharacterization and selection	Before proposing a bioremediation remedy, it is necessary to conduct adequate remedial investigation work to characterize the extent of the contamination. Site selection procedures include determining the horizontal and vertical extent of contamination, defining parameters and sampling locations, and describing sampling and analysis methods.	[40]
Metal ions	Metals are essential for bacteria and fungi, but excessive amounts inhibit cell metabolism. Degradation rates are influenced by metal compounds on both a direct and indirect basis.	[41]
Microorganisms	High concentrations of some toxic compounds can harm microorganisms and slow decontamination process. Toxicity varies with the toxicant, concentration, and microorganisms exposed.	[42]

**Table 2:** Limitations of various bioremediation techniques.

Methods	Limitation	Reference
Biopile	The extent of weathering can change the chemical make-up by making the materials more hydrophobic, which limits the potential of the biopiling method for biodegradation.	[86]
Windrows	The major limitation in studying windrows *in situ* is probably knowing where and when they will emerge. Although it is possible to forecast some sub-mesoscale convergences, it is still difficult to predict where and when litter windrows would form because of the additional uncertainty brought on by the dependency on litter loading.	[87]
Land Farming	This method has the drawback that the objectives specified in the constraint set must be strictly upheld; if they are not, the issue will appear to be insurmountable. Fresh organic waste can be troublesome since it can occasionally lead to anoxic conditions, which are hazardous to plant development. To preserve the quality of pre-existing soils, it is advisable to refrain from adding more organic material over years.	[88]
Bioreactor	The primary limitation to employing membrane bioreactors (MBR) at such high concentrations of mixed liquid suspended solids (MLSS) appears to be very low to zero oxygen transfer efficiency reported when using traditional diffused aeration systems (such as fine and coarse bubble diffusers). This suggests that a deeper understanding is required of the constraints imposed by traditional bubble diffusers (measured in terms of the alpha factor) under that specific combination of operational parameters (high MLSS).	[89]
Intrinsic *in situ* bioremediation	The site has to have very permeable soil for *in situ* bioremediation, which is the main limitation of *in situ* bioremediation.	[90]
Bioventing	This technique’s disadvantage is that it only works at the deepest levels of the contaminated soil ecosystem.	[91]
Phytoremediation	Phytoremediation, such as phytoextraction and rhizodegradation, is used to remediate the polluted soil in the superficial layers of the soil. This approach could be time-consuming and may not be able to eliminate all the contaminants.	[85]

**Table 3:** Potentially hazardous organic and inorganic pollutants and their degrading microbes (bacteria, fungi, and algae).

Substrate	Compound	Microorganisms	References
Organic substrate	Chlorobenzenes	*P. putida* (GJ31)	[106]
	N, N-dimethyl-pphenylenediamine	*Klebsiella pneumonia* (RS-13)	[107]
	Polycyclic aromatic hydrocarbons	*Burkholderia* sp., *Myceliophthorathermophila*	[108]
	Remazol Black B	*Kluyveromyces marxianus (IMB3)*	[109]
	Sulfonate benzene	*A. radiobacter* (S2)	[110]
	4,4 dibromodiphenyl ether	*Phanerochaete chrysosporium*	[111]
	Aromatic hydrocarbons	*Acinetobacter* sp., *Microbacterium* sp., *Pseudomonas* sp. and *Ralstonia* sp.	[112]
	Phenol	*Alcaligenes odorans*, *Corynebacterium propinquum*, *B. subtilis*, and *P. aeruginosa*	[113]
	Toluene and its derivatives	*P. putida* (F1), *Penicillium chrysogenum*	[114]
	Methyl parathion and chlorpyrifos	*Acinetobactor* sp., *Pseudomonas* sp., *Enterobacter sp.* and *Photobacterium* sp.	[115]
	Endosulfan	*Bacillus*, *Staphylococcus*	[116]
	Azo dyes effluents	*Exiguobacterium indicum*, *B. cereus*, *E. aurantiacums* and *A. baumanii*	[117]
	Vat dyes	*B. firmus*, *Staphylococcus aureus*, *B. macerans*, and *K. oxytoca*	[118]
	Oil-based based paints	*B. subtilis* strain NAP1, NAP2, NAP4	[119]
	Crude oil	*Aspergillus niger*, *Candida krusei*, *C. glabrata*, and *Saccharomyces cerevisiae*	[120]
	Diesel oil	*P. cepacia*, *B. coagulans*, *B. cereus*, *B. cereus A* and *Serratia ficaria*	[121]
	Oils	*Alcaligenes odorans*, *Corynebacterium propinquum*, *P. aeruginosa* and *Fusarium* sp.	[122]
Inorganic substrate	Heavy metals, mercury nickel and lead	*Saccharomyces cerevisiae* and, *Cunninghamella elegans*	[123]
	Cr^6+^	*Pseudomonas putida*	[124]
	Cobalt, chromium, copper, and lead	*Lysinibacillus sphaericus* CBAM5	[125]
	Cadmium	*A. versicolor*, *Paecilomyces* sp., *A. fumigatus*, *Paecilomyces* sp., *Terichoderma* sp. and *Cladosporium* sp.	[126]
	Uranium, copper, nickel, chromium	*P. aeruginosa*, *Aeromonas* sp.	[127]
	Lead, chromium, and cadmium	*Aerococcus* sp., *Rhodopseudomonas palustris*	[128]
	Hg^2+^	*Cyclotella cryptica*, *Chlamydomonas reinhardtii*, *Pseudochlorococcum typicum*, *Spirogyra hyaline*	[129]
	Cr_2_O_7_^22^	*Chlorella* spp. *Spirulina* sp. (HD-104)	[130]
	Cr^51^	*Spirulina* sp., *Ulothrix tenuissima* and *C. reinhardtii*	[131]
	Pb^21^	*Oscillatoria laete-virens*, *Arthrospira platensis*, *Pseudochlorococcum typicum* and *Spirogyra insignis*	[132]

## Data Availability

Data that support these findings available within the article.
